# Phenotype-Guided Outpatient Levosimendan as a Bridge-to-Transplant in Low-Output Advanced Heart Failure: A Single-Center Cohort

**DOI:** 10.3390/jpm15100473

**Published:** 2025-10-02

**Authors:** Ricardo Carvalheiro, Ana Raquel Santos, Ana Rita Teixeira, João Ferreira Reis, António Valentim Gonçalves, Rita Ilhão Moreira, Tiago Pereira da Silva, Valdemar Gomes, Pedro Coelho, Rui Cruz Ferreira

**Affiliations:** 1 Department of Cardiology, Unidade Local de São José/Hospital de Santa Marta, R. de Santa Marta 50, 1169-024 Lisbon, Portugalantonio.goncalves4@ulssjose.min-saude.pt (A.V.G.);; 2 Department of Cardiac Surgery, Unidade Local de São José/Hospital de Santa Marta, R. de Santa Marta 50, 1169-024 Lisbon, Portugal

**Keywords:** advanced heart failure, phenotype-guided therapy, heart transplantation, bridge-to-transplant, levosimendan, peri-transplant outcomes

## Abstract

**Background**: Advanced heart failure (HF) carries high morbidity and mortality, and deterioration on the heart transplantation (HT) waiting list remains a major challenge. Intermittent outpatient levosimendan has been proposed as a bridge strategy, but the optimal regimen and its impact on peri-transplant outcomes remain uncertain. Within a personalized-medicine framework, we targeted a low-output/INTERMACS 3 phenotype and operationalized an adaptable, protocolized levosimendan pathway focused on perfusion/congestion stabilization to preserve transplant candidacy. **Methods**: We conducted a single-center, retrospective cohort study of 25 consecutive adults actively listed for HT between 2019 and 2024, treated with a standardized outpatient program of a 14-day interval of 6 h intravenous levosimendan infusions (target 0.2 μg/kg/min infusions) continued until transplant. Personalization in this program was operationalized through (i) phenotype-based eligibility (low CI and elevated filling pressures despite GDMT), (ii) predefined titration and safety rules for blood pressure, arrhythmias, and renal function, and (iii) individualized continuation until transplant with nurse-supervised monitoring and review of patient trajectories. Baseline characteristics, treatment exposure and safety, changes in hospitalizations and biomarkers, and peri-transplant outcomes were analyzed. **Results**: Patients were predominantly male (68%), with a mean age of 47.9 ± 17.5 years and severe LV dysfunction (LVEF 30.6 ± 9.8%). Median treatment duration was 131 days (IQR 60–241). No infusions required discontinuation for hypotension or arrhythmia, and no adverse events were directly attributed to levosimendan. Two patients (8%) died on the waiting list, both unrelated to therapy. During treatment, HF hospitalizations decreased significantly compared with the previous 6 months (48% vs. 20%, *p* = 0.033), renal function remained stable, and NT-proBNP trended downward. Of the 23 patients transplanted, two (9%) underwent urgent HT during decompensation. Post-transplant, vasoplegia occurred in 26% (n = 6 of 23), and 30-day mortality was 9% (n = 2 of 23). **Conclusions**: By defining the target phenotype, therapeutic goals, and adaptation rules, this study shows how a standardized but flexible outpatient levosimendan regimen can function as a personalized bridge strategy for low-output advanced HF. The approach was associated with fewer hospitalizations, stable renal function, and acceptable peri-transplant outcomes, and merits confirmation in multicenter cohorts with attention to patient heterogeneity and treatment effect refinement.

## 1. Introduction

Advanced heart failure (HF) carries substantial symptom burden, recurrent hospitalizations, and high mortality despite contemporary guideline-directed therapy. Among eligible patients, there is clinical consensus that heart transplantation (HT) improves survival and quality of life, yet deterioration and death on the waiting list remain clinically meaningful, underscoring the need for strategies that stabilize low-output phenotypes while preserving transplant candidacy [1,2,3,4].

Continuous intravenous inotropes can support perfusion, but their prolonged use often requires central access and is complicated by infections and other adverse effects, making ambulatory, catheter-free options attractive where feasible [5,6]. Levosimendan, a calcium sensitizer and inodilator with a long-acting metabolite, has been explored as an intermittent outpatient therapy, albeit with different administration strategies [7,8]. Across randomized and prospective studies (LEVO-Rep, LION-HEART, and LAICA), pulsed or periodic levosimendan reduced natriuretic peptides and HF hospitalizations, with signals (though not definitive evidence) toward improved outcomes [9,10,11]. Most recently, the multinational LeoDOR trial tested repetitive levosimendan in patients with advanced HF during the vulnerable post-discharge period. The study, underpowered due to COVID-19, found no benefit on the global rank primary endpoint and even a signal toward higher clinical events with levosimendan, highlighting uncertainty regarding the optimal timing, dosing, and patient selection for intermittent levosimendan in advanced HF [12].

Building on this body of work, our group previously described a pragmatic, nurse-supervised outpatient program delivering intermittent 6 h infusions that was associated with fewer HF admissions and improved functional status [13].

For patients actively awaiting HT, evidence is emerging. A recent large multicenter Spanish registry included more than 300 HT candidates treated with repetitive ambulatory levosimendan, making it the largest cohort described to date. In that study, therapy was used as a bridge in highly symptomatic patients, with variable dosing strategies (24 h or shorter infusions, with different intervals between sessions), and was associated with stabilization of clinical status and maintenance of transplant eligibility [14]. However, the heterogeneity of regimens across centers highlights the need for more standardized approaches.

Reflecting this evolving evidence base, the 2024 ISHLT Guidelines for the Evaluation and Care of Cardiac Transplant Candidates provide a Class IIb, Level B-R recommendation for periodic administration of levosimendan (either as 24–48 h continuous infusions or shorter intermittent infusions) in advanced HF patients with evidence of organ hypoperfusion awaiting HT, with the aim of improving hemodynamic status and congestion, enabling optimization of GDMT, reducing symptoms and hospitalizations, and improving quality of life [2].

At the same time, peri-transplant clinicians often worry that the vasodilatory profile of levosimendan could be a risk factor for vasoplegia—a frequent complication of cardiac surgery and heart transplant. While this is a biologically plausible concern, data in cardiac surgery do not show a definitive increase in vasopressor-resistant vasoplegic syndrome attributable to levosimendan, highlighting the need for context-specific, real-world evidence in HT pathways [15,16,17].

Despite these signals, the optimal outpatient dosing, monitoring, and patient-selection framework for candidates on the HT waiting list is not established, and comparative data across real-world protocols are limited. From a personalized-care perspective, there is a need to define how a standardized yet adaptable levosimendan pathway can be integrated with individual risk profiles, including hemodynamics, renal function, arrhythmic risk, and hospitalization burden, to keep patients transplant-eligible and out of the hospital.

Within the field of personalized cardiology and advanced heart failure, emphasis has shifted from disease labels to treatable traits and clinical phenotypes, with a focus on tailoring therapy to hemodynamic profiles, dynamic risk, and patient-centered goals. Our program reflects this paradigm by targeting the low-output/INTERMACS 3 phenotype and embedding structured titration, safety monitoring, and individualized continuation until transplant.

Against this background, we report our single-center experience with a biweekly 6 h outpatient levosimendan protocol as a bridge to HT. The primary aim of this study was to evaluate the safety and effectiveness of a standardized, every-14-day levosimendan infusion protocol as a bridge-to-transplant strategy in advanced heart failure patients with a low-output/INTERMACS 3 phenotype. We describe baseline characteristics, treatment exposure and safety, short-term effects on hospitalizations and biomarkers, and peri-transplant outcomes including vasoplegia and 30-day mortality. 

## 2. Methods

### 2.1. Study Design and Population

We performed a single-center, retrospective cohort study of consecutive adults with advanced heart failure (HF) who were actively listed for heart transplantation (HT) and treated with intermittent outpatient levosimendan as bridge therapy at our tertiary HF-transplant center between January 2019 and December 2024. All patients fulfilled HT indications according to the European Society of Cardiology (ESC) heart failure guidelines and the International Society for Heart and Lung Transplantation (ISHLT) listing criteria.

### 2.2. Eligibility for Levosimendan Administration

Eligibility was guided by a phenotype-based approach, focusing on patients with advanced HF and low-output physiology while integrating both objective hemodynamic parameters and clinical risk markers. Patients who met all of the following criteria were considered eligible:

- Age > 18 years;
- Left ventricular ejection fraction (LVEF) < 40%;
- Persistent symptoms of HF despite guideline-directed medical therapy;
- Persistently elevated NT-proBNP levels;
- At least one HF hospitalization in the previous 6 months or severe impairment of exercise capacity (peak oxygen uptake ≤ 12–14 mL/kg/min if under beta-blocker therapy);
- Low-output phenotype (e.g., low Cardiac Index with elevated PCWP and hypotension despite GDMT) consistent with INTERMACS 3 functional profiles.

Patients receiving levosimendan as supportive therapy for advanced HF not listed for transplantation were excluded from this analysis. Final inclusion was determined by the advanced HF team based on clinical judgment, integrating the phenotype features described (low-output physiology with congestion despite GDMT), rather than strict quantitative thresholds. Patients who were screened but not included were not systematically recorded.

### 2.3. Intervention: Intermittent Outpatient Levosimendan Program

This protocol has been previously described by our group and was equally applied in this cohort [13].

Levosimendan was administered every two weeks as a 6 h intravenous infusion in the outpatient unit, without a bolus, with a target dose of 0.2 μg/kg/min. Treatment was regularly administered until a suitable donor became available or until meaningful clinical improvement rendered continuation unnecessary.

During the first cycle, infusion began at 0.05 μg/kg/min, increased to 0.1 μg/kg/min after 2 h if well tolerated, and to 0.2 μg/kg/min after a further 2 h. Subsequent sessions targeted 0.2 μg/kg/min if tolerated.

Each infusion was supervised by the nursing team following a standardized protocol. Before the start of treatment, patients underwent assessment of vital signs, body weight, venous access, venous blood gas (including potassium), and routine laboratory testing. During infusion, vital signs were monitored at 30 min intervals (or more frequently if clinically required), and urine output was recorded. Patient education was reinforced at each session, with emphasis on self-monitoring, adherence, recognition of decompensation triggers, dietary advice, and management of comorbidities. Potassium supplementation was administered when levels were <3.5 mmol/L, to maintain values between 4–5 mmol/L both at baseline and at the end of infusion. At the end of treatment, venous blood gas analysis, body weight, and vital signs were repeated, and venous access was removed.

The attending physician was contacted if patients developed hypotension or arrhythmias. In cases of symptomatic hypotension or SBP < 75 mmHg, the dose was reduced to 0.1 μg/kg/min, with further reduction to 0.05 μg/kg/min or discontinuation if symptoms persisted. Guideline-directed medical therapy (GDMT) was maintained throughout the program. Diuretic dosages were adjusted as per clinical signs of congestion or volume overload, in line with standard practice, and were not altered specifically because of levosimendan infusion.

### 2.4. Personalization Framework

Eligibility was focused on a low-output phenotype, broadly defined to integrate both hemodynamic parameters (low CI, elevated filling pressures) and clinical characteristics such as hypotension, renal vulnerability, and recent decompensation, with final inclusion determined at the discretion of the advanced heart failure team. The protocol standardized how levosimendan was delivered (q14-day, 6 h, no bolus; target 0.2 µg/kg/min with stepwise uptitration), while personalization occurred through (i) predefined titration/hold rules for SBP, arrhythmias, potassium, and renal function; (ii) individualized continuation to HT based on stability and patient preference; and (iii) nurse-supervised session monitoring (vitals q30 min, urine output, electrolytes) with same-day physician escalation thresholds. We reviewed N-of-1 trajectories (admissions, labs, tolerance) to adapt future sessions.

### 2.5. Data Collection and Time Points

Baseline assessments (clinical data, laboratory tests, ECG, transthoracic echocardiography, right-heart catheterization, and CPET) were obtained at program entry. Pre-transplant (or last available) laboratory values were collected immediately before HT. Post-HT data included ischemia time, length of stay, days on aminergic support, and 30-day outcomes.

### 2.6. Outcomes and Definitions

- Exposure metrics: duration of levosimendan therapy (days), number of sessions, and days from last session to HT.
- Safety metrics: infusion discontinuation, severe hypotension/arrhythmias, deaths while on program.
- Effectiveness during bridge: change in HF hospitalizations comparing the 6 months before program start with the treatment period; changes in creatinine, eGFR, NT-proBNP, and troponin from baseline to pre-HT.
- Peri-transplant outcomes: ischemia time, post-HT length of stay, days of aminergic support, vasoplegia, and 30-day mortality.
- Vasoplegia was predefined as a requirement for high-dose vasopressors (≥0.5 μg/kg/min norepinephrine equivalents) despite adequate fluid resuscitation.

### 2.7. Statistical Analysis

Continuous variables were checked for normality (visual inspection of histograms and Shapiro–Wilk test). Normally distributed data are reported as mean ± SD and compared within-patient using paired *t*-tests; non-normal data are reported as median (IQR) and compared using Wilcoxon signed-rank tests. Paired counts (e.g., number of HF hospitalizations pre- vs. during the program) were compared with the Wilcoxon signed-rank test. Categorical variables are presented as n (%) and compared using McNemar’s test for paired proportions when appropriate, or Fisher’s exact test for between-group. A two-sided *p* < 0.05 was considered statistically significant. Analyses were performed in IBM SPSS Statistics (Version 29, IBM Corp., Armonk, NY, USA).

## 3. Results

### 3.1. Baseline Characteristics

We included 25 patients with advanced heart failure treated with intermittent outpatient levosimendan as bridge therapy to heart transplantation. The mean age was 47.9 ± 17.5 years, and 68% were male.

**Figure 1:**
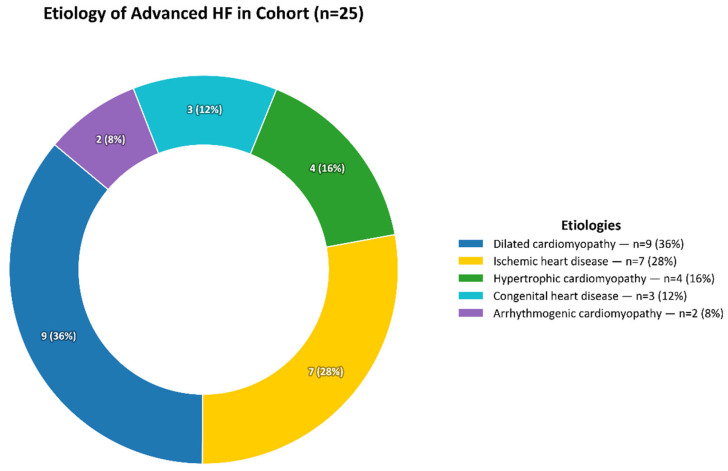
Etiology of advanced heart failure in the study cohort.

The etiology of heart failure was heterogeneous, with dilated cardiomyopathy (36%) being the most common, followed by ischemic heart disease (28%), hypertrophic cardiomyopathy (16%), congenital heart disease (12%), and arrhythmogenic cardiomyopathy (8%) (Figure 1).

As summarized in Table 1, comorbidities were frequent, particularly atrial fibrillation and chronic kidney disease (both 48%). Most patients were in NYHA class III (36%) or IV (48%) at baseline. Regarding renal function, 52% had an estimated GFR ≥ 60, 40% between 30–59, and 8% < 30 mL/min/1.73 m^2^. NT-proBNP levels were markedly elevated, with a median of 4336 pg/mL [IQR 1721–7953], reflecting severe neurohormonal activation and advanced disease status.

All patients were receiving guideline-directed medical therapy: RAAS inhibition/ARNI and beta-blockers (100%), mineralocorticoid receptor antagonists (96%), and SGLT2 inhibitors (84%). Ivabradine and digoxin were less common (16% and 12%). The median furosemide equivalent dose was 80 mg (IQR 50-160). Device therapy was also frequent, with 88% having an ICD and 24% a CRT device.

### 3.2. Echocardiographic, Hemodynamic, and Functional Characteristics

Echocardiography showed advanced structural and functional impairment, with a mean LVEF of 30.6 ± 9.8%, severely abnormal global longitudinal strain (median −8.6% [IQR −10.4–2.5]), and marked left atrial enlargement (LAVi 59.2 ± 19.6 mL/m^2^). Right ventricular systolic function was borderline (TAPSE 16.0 ± 3.5 mm).

Hemodynamics revealed a low cardiac output (CI 2.0 ± 0.4 L/min/m^2^, CO 3.8 ± 0.9 L/min) with elevated filling pressures (PCWP 20.1 ± 7.3 mmHg, CVP 11.1 ± 6.0 mmHg), and a mean CPO of 0.25 ± 0.08 W, consistent with severely impaired global cardiac pumping capacity. There were also signs of pulmonary hypertension (mPAP 30.0 ± 8.3 mmHg), with mildly elevated PVR (2.7 ± 1.5 WU), consistent with pre- and post-capillary pulmonary hypertension, but preserved transplant eligibility.

Derived indices showed a mean PAPi of 3.0 ± 2.3 and an RVSWI of 8.3 ± 4.5 g·m/m^2^, consistent with borderline-to-preserved RV performance.

Functional capacity was severely impaired: mean peak VO_2_ 13.1 ± 2.9 mL/kg/min (44 ± 8% predicted) and VE/VCO_2_ slope 42.9 ± 11.0. Full parameters are shown in Table 2.

### 3.3. Levosimendan Exposure and Safety

The median treatment duration was 131 days (IQR 60–241), corresponding to 9 sessions (IQR 5–13). The median interval between the last infusion and transplantation was 8 days (IQR 2–11).

At baseline, patients had a reduced blood pressure profile (systolic blood pressure 92.3 ± 9.9 mmHg, diastolic blood pressure 59.0 ± 9.2 mmHg, mean blood pressure 71.0 ± 8.5 mmHg, heart rate 67.2 ± 14.1 bpm). Despite this, and despite a high prevalence of prior ventricular arrhythmias, no severe hypotension or malignant arrhythmias requiring discontinuation occurred. Overall, therapy was well tolerated.

Two deaths (8%) occurred while patients were still on levosimendan therapy in the waiting list, both considered unrelated to treatment: one from an arrhythmic storm eight days after the last infusion, and one from progressive cardiogenic shock requiring urgent left ventricular mechanical support. Of the remaining 23 patients who underwent transplantation, 2 (9%) were transplanted during an urgent admission for decompensated heart failure.

### 3.4. Treatment Outcomes

**Figure 2:**
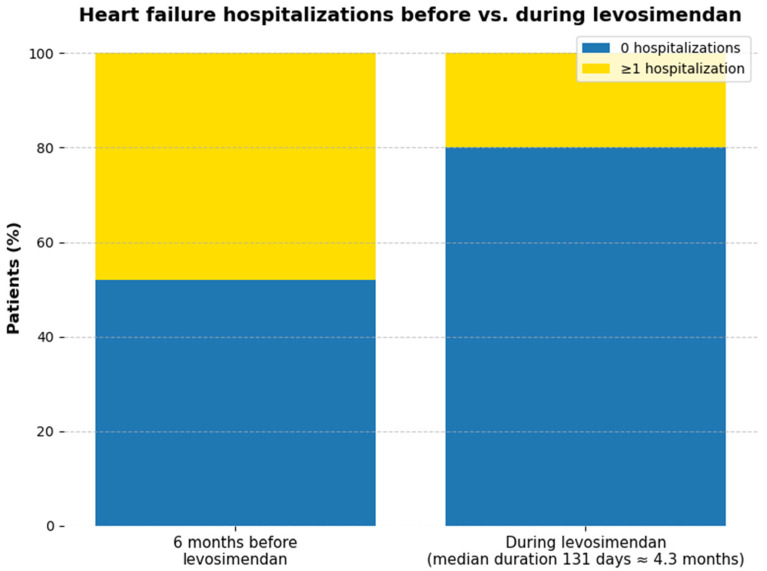
Frequency of hospitalizations for decompensated heart failure in the 6 months prior to levosimendan initiation and during intermittent levosimendan therapy.

Hospitalizations for decompensated HF were fewer during levosimendan therapy than in the preceding 6 months (Figure 2) In total, 12 hospitalizations occurred before program initiation (48% of patients, corresponding to 0.079 events per patient-month), compared with 5 hospitalizations during levosimendan (20% of patients, 0.035 events per patient-month). This represents an absolute reduction in 7 events and a 28% decrease in the proportion of patients hospitalized (*p* = 0.033). Given the small sample size, these findings are best interpreted descriptively; *p*-values are reported as exploratory only.

Renal function remained stable, as assessed by creatinine levels (1.38 ± 0.38 vs. 1.33 ± 0.36 mg/dL, *p* = 0.253) and eGFR (62.2 ± 26.1 vs. 63.3 ± 23.4 mL/min/1.73 m^2^, *p* = 0.645). NT-proBNP showed a non-significant downward trend (4336 pg/mL [IQR 1721–7953] vs. 3113 pg/mL [IQR 1197–6181]; *p* = 0.232). Troponin remained unchanged (32 ng/L [IQR 15–56] vs. 29 ng/L [IQR 12–55]; *p* = 0.872).

### 3.5. Peri-Transplant Outcomes

The mean ischemia time was 195.7 ± 52.5 min, and the mean post-transplant hospital stay was 21.7 ± 4.9 days. The median duration of vasopressor or inotropic support was 3 days (IQR 3–4).

Vasoplegia occurred in 6 patients (26%). Two patients (9%) died within 30 days after transplantation, both due to severe primary graft dysfunction. Exploratory analyses did not show a significant association between duration of levosimendan exposure and vasoplegia (r = 0.36, *p* = 0.096). Associations with graft dysfunction could not be reliably evaluated because of the very small number of events. For context, among all 56 heart transplants performed at our institution during the same period (2019–2024), the 30-day mortality was 12.5% (7 of 56), indicating that outcomes in this levosimendan-treated cohort were within the expected institutional range.

**Figure 3:**
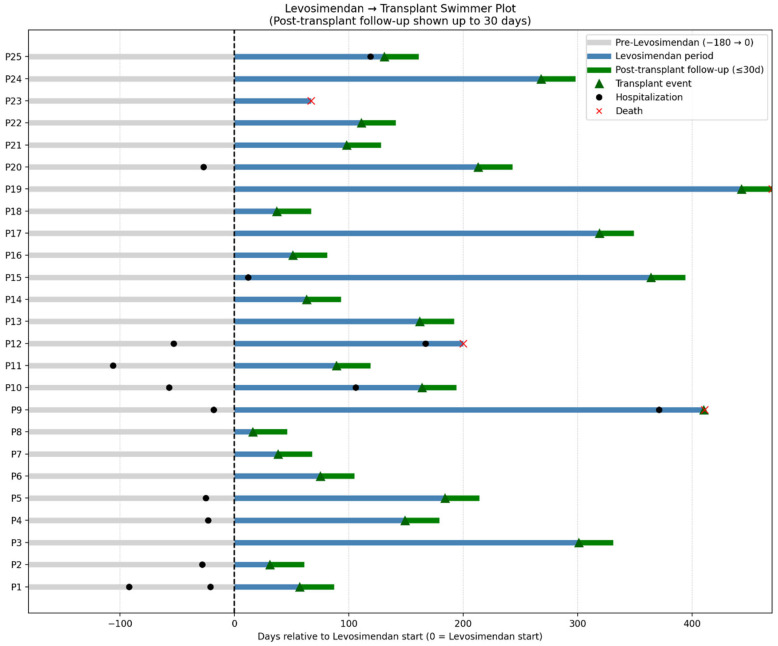
Swimmer plot showing individual patient trajectories from six months before levosimendan initiation until transplantation and 30-day follow-up. Each bar represents one patient, with the duration of levosimendan therapy, the time to transplantation, and post-transplant outcome (alive or dead at 30 days).

The individual trajectories from six months before levosimendan initiation through transplantation and 30-day post-transplant follow-up are shown in Figure 3. This swimmer plot illustrates the duration of levosimendan therapy, timing of transplantation, and early post-transplant outcomes, including survival or death within 30 days.

## 4. Discussion

In this single-center experience of actively listed HT candidates treated with a standardized, 14-day interval outpatient levosimendan regimen, therapy was well tolerated. Despite low baseline blood pressure and frequent prior ventricular arrhythmias, no infusions had to be discontinued due to severe hypotension or arrhythmia, and no adverse events were directly attributable to levosimendan.

Two patients died on the waiting list, under levosimendan treatment. Although both events were adjudicated as unrelated to infusion, one death occurred eight days after the last administration due to an arrhythmic storm. While causality cannot be inferred, we acknowledge this temporal proximity. Importantly, no proarrhythmic signals were observed during infusions, and both deaths were judged more consistent with an advanced disease trajectory than with treatment effect.

Effectiveness signals paralleled findings from several prior trials of intermittent levosimendan in advanced HF. Hospitalizations for decompensated HF were significantly reduced during the treatment period, renal function remained stable, and NT-proBNP trended downward. Preserving renal function and reducing admissions are clinically relevant in the transplant pathway, as both help sustain eligibility and avoid escalation to mechanical support. These observations are consistent with outpatient studies such as LEVO-Rep, LION-HEART, and LAICA, where reductions in natriuretic peptides and hospitalizations were consistently reported. In contrast, the recent multinational LeoDOR trial, which evaluated repetitive levosimendan in the post-discharge vulnerable phase, did not show improvement in its global rank primary endpoint and even signaled more clinical events in the levosimendan arm. Differences in timing, patient selection, and trial power likely explain the discordance, underscoring the importance of context-specific evidence when applying intermittent levosimendan strategies.

Our observations also complement those of the large multicenter Spanish cohort, the largest experience of ambulatory levosimendan in HT candidates. That registry, though based on heterogeneous dosing regimens and intervals, showed that repetitive levosimendan could maintain clinical stability and transplant candidacy in high-risk patients. Notably, the most frequent regimens were fixed doses of 6.25–12.5 mg per session or 24 h infusions at 0.1 μg/kg/min, while only ~15% of patients received the same 6-h, 0.2 μg/kg/min 14-day interval protocol applied in our study. In this sense, our program provides a uniform and reproducible model of a regimen that is underrepresented in real-world practice.

At the same time, our protocol is closely aligned with dosing schemes evaluated in prior prospective trials. Both LION-HEART and LevoRep tested 6 h infusions at 0.2 μg/kg/min every two weeks for fixed durations of 12 and 6 weeks, respectively, and one arm of LeoDOR employed the same regimen, whereas its second arm evaluated 24 h infusions at 0.1 μg/kg/min every three weeks. By contrast, the LAICA trial adopted a monthly 24 h infusion at 0.1 μg/kg/min over one year. Our contribution builds on these studies by extending the biweekly 6-h, 0.2 μg/kg/min regimen indefinitely until transplantation, embedding predefined titration rules and nurse-supervised monitoring to ensure safety over prolonged use. This combination of continuity with established regimens and adaptation to the transplant pathway highlights a practical, standardized, yet flexible bridge-to-transplant strategy.

Peri-transplant outcomes in our patients were in line with published experience. Vasoplegia occurred in roughly one quarter of patients, and 30-day mortality was under 10%. While the low absolute numbers preclude meaningful comparison with larger series, levosimendan administration did not appear to adversely affect short-term post-transplant outcomes in this high-risk cohort.

From a personalized-care perspective, this protocol illustrates how a standardized regimen can be tailored to individual patient needs. Fixed session cadence, predefined titration, and structured monitoring were applied consistently, while therapy was prolonged until transplant when clinically indicated. In practice, personalization was expressed through three main elements: (i) targeting a low-output/INTERMACS 3 phenotype with congestion; (ii) addressing treatable traits such as perfusion and congestion using pragmatic markers, including CI, PCWP, and renal trends; and (iii) applying dynamic, session-by-session adjustments through nurse-supervised monitoring and patient-specific trajectories. This standardized-yet-adaptable model shows how protocolized hemodynamic tailoring can reduce HF hospitalizations, preserve transplant eligibility, and maintain peri-transplant safety in this high-risk phenotype. The approach also aligns with guideline recommendations suggesting levosimendan may be considered in advanced HF patients awaiting HT to improve hemodynamics, congestion, and clinical stability, while acknowledging that the optimal regimen remains to be defined.

A main strength of this work is the demonstration of a uniform 6 h dosing regimen continued until transplantation, adaptable to individual patient needs. This contrasts with the heterogeneous approaches reported in larger cohorts and suggests a pathway for standardization that still respects personalized risk profiles. Because this pathway avoids long-term central access and relies on nurse-supervised day-unit infusions with simple physiologic triggers, it is implementation-ready and potentially scalable. However, practical barriers such as staffing of outpatient infusion centers, patient transportation, therapy cost, and local resource availability may limit widespread adoption and should be considered when translating this model into routine practice.

Limitations of this study include its retrospective, single-center design, small sample size, absence of a control group, and short post-transplant follow-up. The lack of a comparator arm precludes causal inference, making it impossible to distinguish treatment effects from natural history, regression to the mean, or selection bias. In addition, patients were included by clinical decision in real-world practice, without systematic recording of those screened but excluded, which limits reproducibility. The cohort was relatively young, with high device penetration and contemporary guideline-directed medical therapy, which may restrict generalizability to older or more comorbid populations. Finally, the small number of hospitalization events during therapy prevented robust recurrent-event modeling; instead, hospitalization rates were reported descriptively as events per patient-month. Larger comparative studies are needed to validate these findings and to identify which patients benefit most from this approach.

## 5. Conclusions

In actively listed patients with advanced HF, a personalized, phenotype-guided outpatient levosimendan regimen of 6 h infusions every 14 days at a target 0.2 µg/kg/min, continued until transplantation, was feasible, well tolerated, and not associated with adverse safety signals. During the bridging period, hospitalizations were reduced, renal function was preserved, and NT-proBNP trended downward. Peri-transplant vasoplegia and early mortality occurred within expected ranges, and levosimendan did not appear to negatively influence short-term outcomes after HT. By codifying eligibility, treatable-trait targets, and adaptation rules, this study presents a reproducible personalization pathway for the low-output phenotype.

These findings, in line with prior randomized trials and the Spanish registry, support intermittent levosimendan as a personalized bridge-to-transplant option. Larger multicenter studies are warranted to refine dosing strategies, optimize patient selection, and clarify its role in peri-transplant management.

## Figures and Tables

**Table 1:** Baseline clinical characteristics of the study cohort. Data are presented as mean ± standard deviation (SD), median (interquartile range [IQR]), or n (%), as appropriate. BMI—body mass index; COPD—chronic obstructive pulmonary disease; CKD—chronic kidney disease; VT—ventricular tachycardia; VF—ventricular fibrillation; NYHA—New York Heart Association; GFR—glomerular filtration rate; RAASi—renin–angiotensin–aldosterone system inhibitor; ARNI—angiotensin receptor–neprilysin inhibitor; MRA—mineralocorticoid receptor antagonist; iSGLT2—sodium–glucose co-transporter 2 inhibitor; ICD—implantable cardioverter–defibrillator; CRT—cardiac resynchronization therapy.

	n = 25	
Age in years—mean ± SD	47.9 ± 17.5	
Male—n (%)	17	(68.0)
BMI—mean ± SD	24.7 ± 4.0	
Dilated cardiomyopathy—n (%)	9	(36.0)
Comorbidities		
Hypertension—n (%)	11	(44.0)
Dyslipidemia—n (%)	15	(60.0)
Diabetes Mellitus—n (%)	6	(24.0)
Smoking history—n (%)	7	(28.0)
Atrial Fibrillation—n (%)	12	(48.0)
COPD—n (%)	4	(16.0)
CKD—n (%)	12	(48.0)
Previous sustained VT or VF—n (%)	10	(40.0)
NYHA Class		
II—n (%)	4	(16.0)
III—n (%)	9	(36.0)
IV—n (%)	12	(48.0)
GFR (mL/min/1.73 m^2^)		
> 60—n (%)	13	(52.0)
30–60—n (%)	10	(40.0)
<30—n (%)	2	(8.0)
Usual Medication		
RAASi/ARNI—n (%)	25	(100.0)
Beta blocker—n (%)	25	(100.0)
MRA—n (%)	24	(96.0)
iSGLT2—n (%)	21	(84.0)
Digoxin—n (%)	3	(12.0)
Ivabradine—n (%)	4	(16.0)
Oral anticoagulation—n (%)	13	(52.0)
Furosemide dose—median (IQR)	80	(50–160)
Devices		
ICD—n (%)	22	(88.0)
CRT—n (%)	6	(24.0)

**Table 2:** Echocardiographic, hemodynamic, and cardiopulmonary exercise test (CPET) parameters of the study cohort. Data are presented as mean ± standard deviation (SD) or median (interquartile range [IQR]), as appropriate. LV TDD—left ventricular telediastolic diameter; LV TSD—left ventricular telesystolic diameter; LVEF—left ventricular ejection fraction; LV GLS—left ventricular global longitudinal strain; TAPSE—tricuspid annular plane systolic excursion; E/e′—mitral inflow E wave to mitral annular e′ ratio; LAVi—left atrial volume index; PCWP—pulmonary capillary wedge pressure; RAP—right atrial pressure; sPAP—systolic pulmonary artery pressure; mPAP—mean pulmonary artery pressure; PVR—pulmonary vascular resistance; SVR—systemic vascular resistance; PAPi—pulmonary artery pulsatility index; RAP/PCWP—right atrial pressure to PCWP ratio; RVSWi—right ventricular stroke work index; CPO—cardiac power output; pVO_2_—peak oxygen consumption; % predicted pVO_2_—peak VO_2_ as percentage of predicted value; VE/VCO_2_ slope—minute ventilation/carbon dioxide production slope; RER—respiratory exchange ratio.

	n = 25	
Echocardiographic Parameters		
LV TDD (mm)—mean ± SD	64.6 ± 12.1	
LV TSD (mm)—mean ± SD	53.3 ± 13.5	
LV Ejection Fraction (%)—mean ± SD	30.6 ± 9.8	
LV GLS (-%)—median (IQR)	8.6	(2.5–10.4)
TAPSE (mm)—mean ± SD	16.0 ± 3.5	
E/e′—mean ± SD	13.4 ± 4.3	
LAVi (mL/m^2^)—mean ± SD	59.2 ± 19.6	
Right Heart Catheterization		
Cardiac Index (L/min/m^2^)—mean ± SD	2.0 ± 0.4	
Cardiac Output (L/min)—mean ± SD	3.8 ± 0.9	
PCWP (mmHg)—mean ± SD	20.1 ± 7.3	
CVP (mmHg)—mean ± SD	11.1 ± 6.0	
sPAP (mmHg)—mean ± SD	44.9 ± 12.0	
mPAP (mmHg)—mean ± SD	30.0 ± 8.3	
PVR (WU)—mean ± SD	2.7 ± 1.5	
SVR (dynes·sec/cm^5^)—mean ± SD	1366.2 ± 579.1	
PAPi—mean ± SD	3.0 ± 2.3	
RAP/PCWP—mean ± SD	0.5 ± 0.3	
RVSWi (g·m/m^2^)—mean ± SD	8.3 ± 4.5	
CPO (W)—mean ± SD	0.25 ± 0.08	
CPET		
pVO2 (mL/kg/min)—mean ± SD	13.1 ± 2.9	
% of predicted pVO2—mean ± SD	44.3 ± 8.4	
VE/VCO2 Slope—mean ± SD	42.9 ± 11.0	
RER—mean ± SD	1.0 ± 0.1	

## Data Availability

The data presented in this study are available upon request from the corresponding author. They are not publicly available due to privacy and data protection regulations (Regulamento Geral sobre a Proteção de Dados—[RGPD] (EU)2016/679).
